# Rigid fibrescope Bonfils: use in simulated difficult airway by novices

**DOI:** 10.1186/1757-7241-17-33

**Published:** 2009-07-22

**Authors:** Tim Piepho, Rüdiger R Noppens, Florian Heid, Christian Werner, Andreas R Thierbach

**Affiliations:** 1 University Medical Center of the Johannes Gutenberg-University, Department of Anaesthesiology, Langenbeckstr. 1, Mainz, 55131, Germany; 2 Dept. of Anaesthesia, Intensive Care Medicine, Emergency Medicine and Pain Therapy Klinikum Idar-Oberstein Dr.-Ottmar-Kohler-Str. 2 Idar-Oberstein, 55743, Germany

## Abstract

**Background:**

The Bonfils intubation fibrescope is a promising alternative device for securing the airway. We examined the success rate of intubation and the ease of use in standardized simulated difficult airway scenarios by physicians. We compared the Bonfils to a classical laryngoscope with Macintosh blade.

**Methods:**

30 physicians untrained in the use of rigid fibrescopes but experienced in airway management performed endotracheal intubation in an airway manikin (SimMan, Laerdal, Kent, UK) with three different airway conditions. We evaluated the success rate using the Bonfils (Karl Storz, Tuttlingen, Germany) or the Macintosh laryngoscope, the time needed for securing the airway, and subjective rating of both techniques.

**Results:**

In normal airway all intubations were successful using laryngoscope (100%) vs. 82% using the Bonfils (p < 0.05). In the scenario "tongue oedema" success rate using the Macintosh laryngoscope was 67% and 83% using the Bonfils. In the scenario "decreased cervical range of motion with jaw trismus", success rate using the Macintosh laryngoscope was 84% vs. 76%. In difficult airway scenarios time until airway was secured did not differ between the two devices. Use of Bonfils was rated "easier" in both difficult airway scenarios.

**Conclusion:**

The Bonfils can be successfully used by physicians unfamiliar with this technique in an airway manikin. The airway could be secured with at least the same success rate as using a Macintosh laryngoscope in difficult airway scenarios. Use of the Bonfils did not delay intubation in the presence of a difficult airway. These results indicate that intensive special training is advised to use the Bonfils effectively in airway management.

## Background

Securing the airway with an endotracheal tube (ET) and inflated cuff offers several benefits in the treatment of anaesthetized patients or those in critical condition. Potential benefits include protection against aspiration, application of facilitated assisted or controlled mechanical ventilation and positive end expiratory pressure [1]. Despite recent advances in airway management, the laryngoscope blade as described by Macintosh (commonly referred to as Macintosh blade) remains the so-called "gold standard" for endotracheal intubation [2].

However, endotracheal intubation is occasionally difficult or impossible with a consecutive increasing morbidity and mortality unless oxygenation is immediately established [3]. In out-of hospital situations the use of an alternative device for endotracheal intubation is required by emergency physicians in 0.9 to 2.6% of all cases [4-6]. Supraglottic airway devices are generally accepted as alternatives in cases of failed endotracheal intubation. However, endotracheal intubation has additional advantages and supraglottic airways may fail as well [7-9].

**Figure 1:**
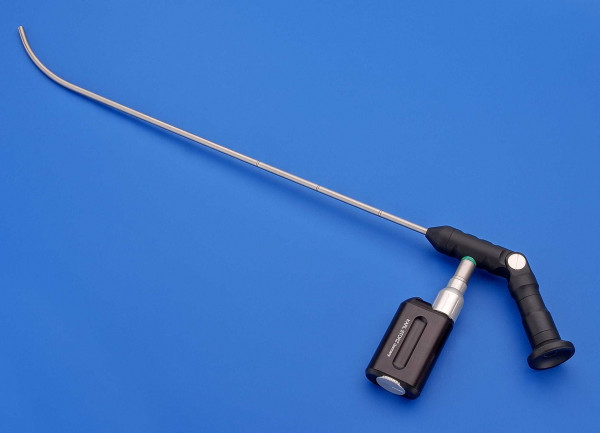
**The Bonfils Intubation Fibrescope (Karl Storz GmbH & Co. KG, Tuttlingen, Germany)**.

The Bonfils intubation fibrescope (Karl Storz GmbH & Co. KG, Tuttlingen, Germany; figure 1) is an alternative to direct laryngoscopy using the Macintosh blade with a potential benefit especially in difficult airway situations [10]. The device has a long thin rigid cylindrical body with a curved tip. A tracheal tube is loaded onto the shaft of the instrument and is pushed into a locking mechanism. After lateralized insertion of the Bonfils into the mouth a jaw thrust maneuver may be used to increase the view into the retropharyngeal space [11]. The rigid fibrescope is advanced into the glottic aperture and the tracheal tube is inserted into the trachea after identifying the vocal cords. The device is not advanced through the vocal cords at anytime.

Recent reports demonstrated that the Bonfils is applicable for tracheal intubation in patients with normal and expected difficult airway [11,12].

The aim of the study was to compare the success rates of intubation of physicians inexperienced in the use of a rigid fibrescope vs. orotracheal intubation performed with help of Macintosh blade in simulated difficult airway scenarios. We hypothesised that physicians inexperienced in the use of a rigid fibrescope succeed orotracheal intubation in simulated difficult airway scenarios.

## Methods

Thirty physicians with at least one year clinical experience in anaesthesiology consented to participate in this study. Prior to the study, each participant completed a questionnaire documenting previous experience with the Macintosh laryngoscope and the Bonfils. All 30 participants had performed a minimum of 100 endotracheal intubations each (range 125 to over 2000) at the time of the investigation using the Macintosh laryngoscope. Only two physicians had prior experience of using the Bonfils in two cases each. Each physician was given a standardized demonstration of the Bonfils by one of the investigators: After a theoretically description of the use, the intubation procedure was practically demonstrated by one of the investigators. The participants did not practice with the instrument prior to the investigation. The Bonfils was used without a camera attached and an extra video screen. View during endotracheal intubation was obtained via the eyepiece of the instrument.

The sequence in which the participants used the devices was randomized. All endotracheal intubations were performed using a standard Magill 8.0 mm tracheal tube (Rüsch, Kernen i.R., Germany) in a Laerdal SimMan manikin (Laerdal, Kent, UK).

The participants were exposed to three different airway scenarios in a randomized order: 1. normal airway, 2. tongue oedema and 3. decreased cervical range of motion with jaw trismus.

The primary endpoints were the success rate of endotracheal intubation and the required time to perform this. Endotracheal intubation attempt without interposed mask ventilation should not exceed 30–40 s [1]. Therefore, failed intubation was defined as an attempt in which endotracheal intubation was not successful or required more than 40 s to perform.

Time was measured from insertion of the blade or the Bonfils until the ET was placed and the first ventilation with a self-inflating bag was successfully performed.

At the end of each scenario, every participant rated the ease of use of the devices on a visual analogue scale (from 0 = extremely difficult to 10 = extremely easy).

### Statistical Analyses

Data are reported as mean ± SEM. Duration of endotracheal intubation was analyzed using one-way analyses of variance and post hoc Bonferroni test. Nonparametric data were analyzed by one-way analyses of variance on ranks (GraphPad Prism version 5.00 for Mac, GraphPad Software, San Diego California USA). Data for successful endotracheal intubation were analyzed using the X^2 ^Test. Differences were considered statistically significant at an error probability < 0.05.

## Results

### Normal airway

**Figure 2:**
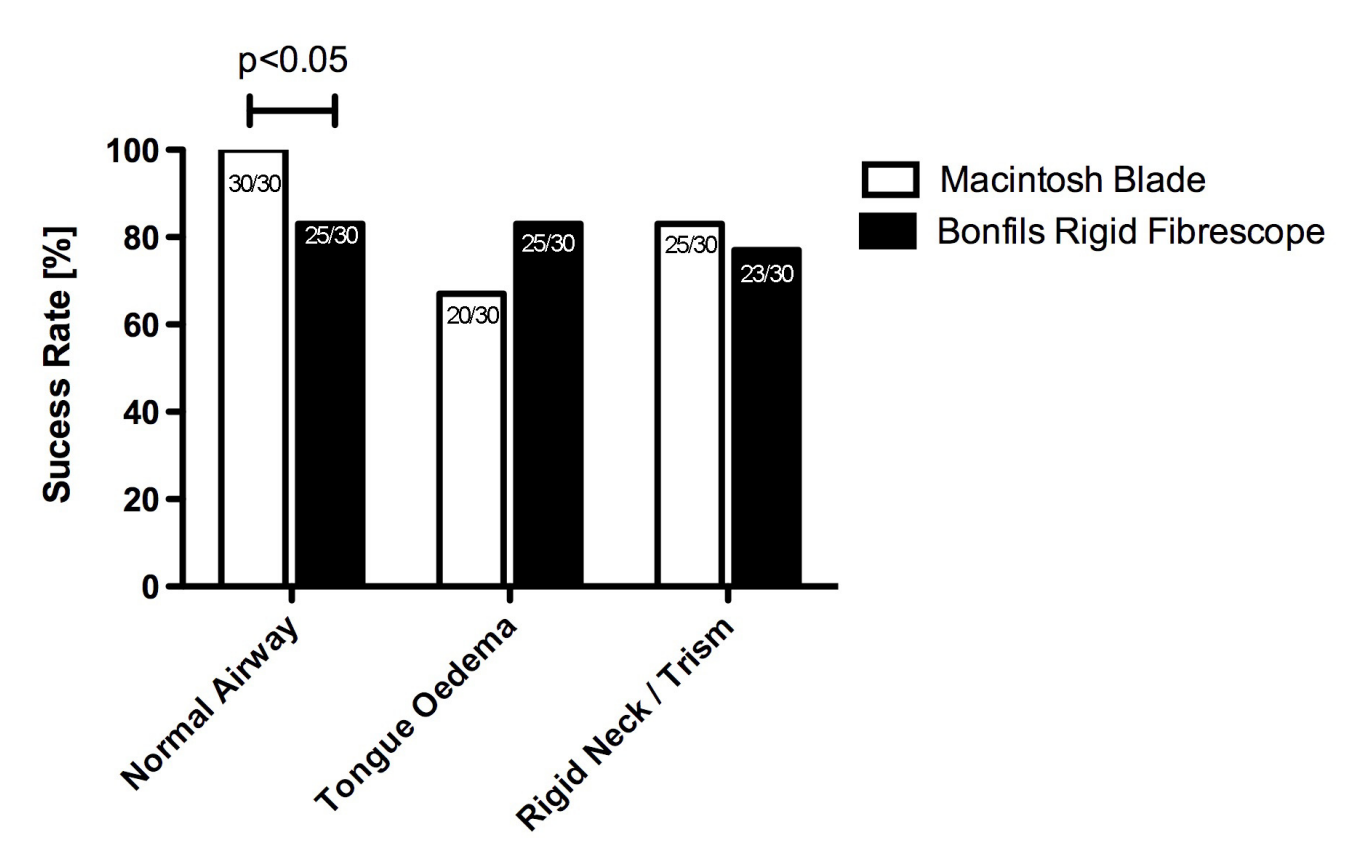
**Successful intubation for different airway scenarios**.

**Figure 3:**
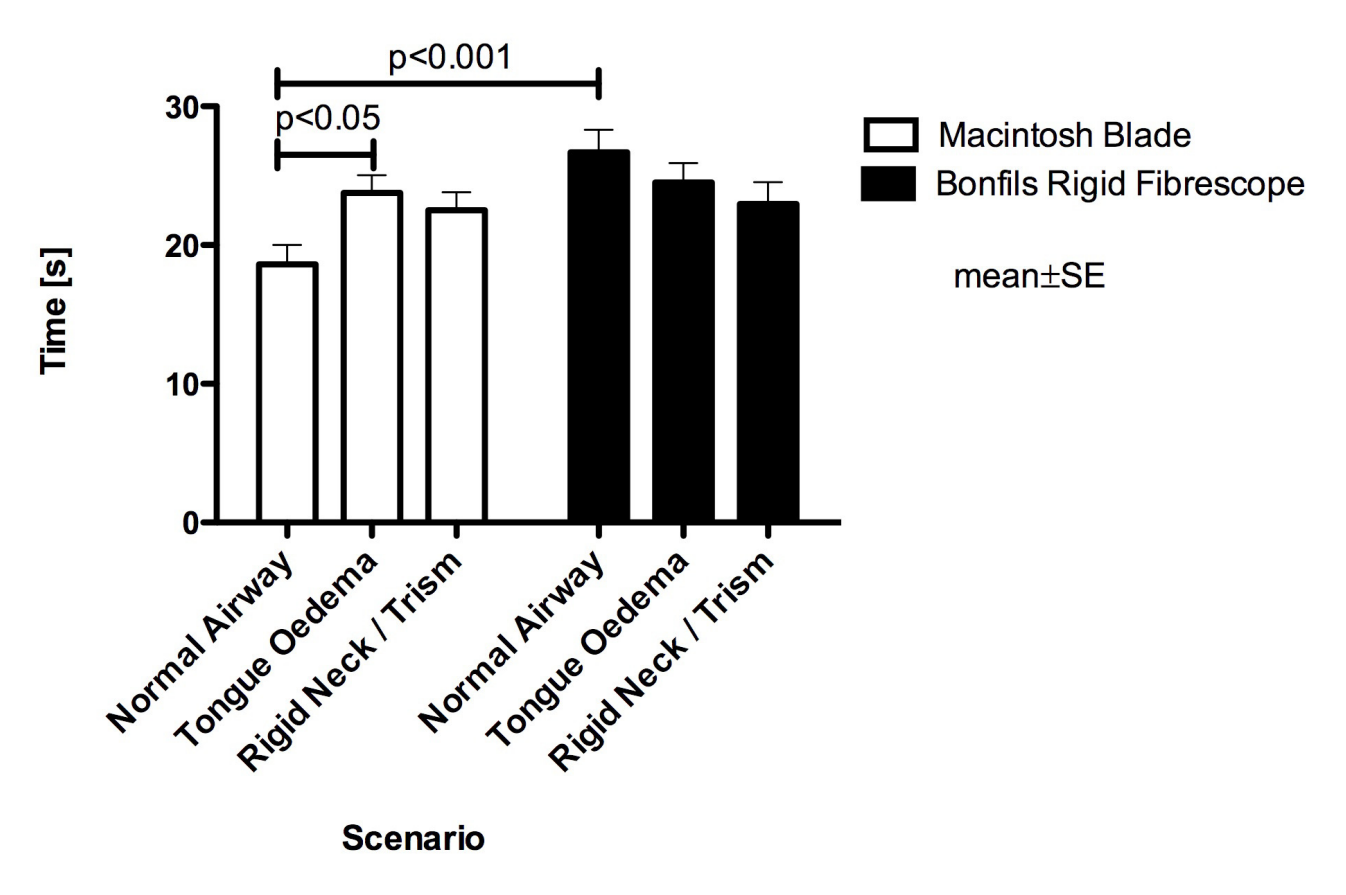
**Time until airway was secured using Bonfils and Macintosh laryngoscope in different airway scenarios**.

**Figure 4:**
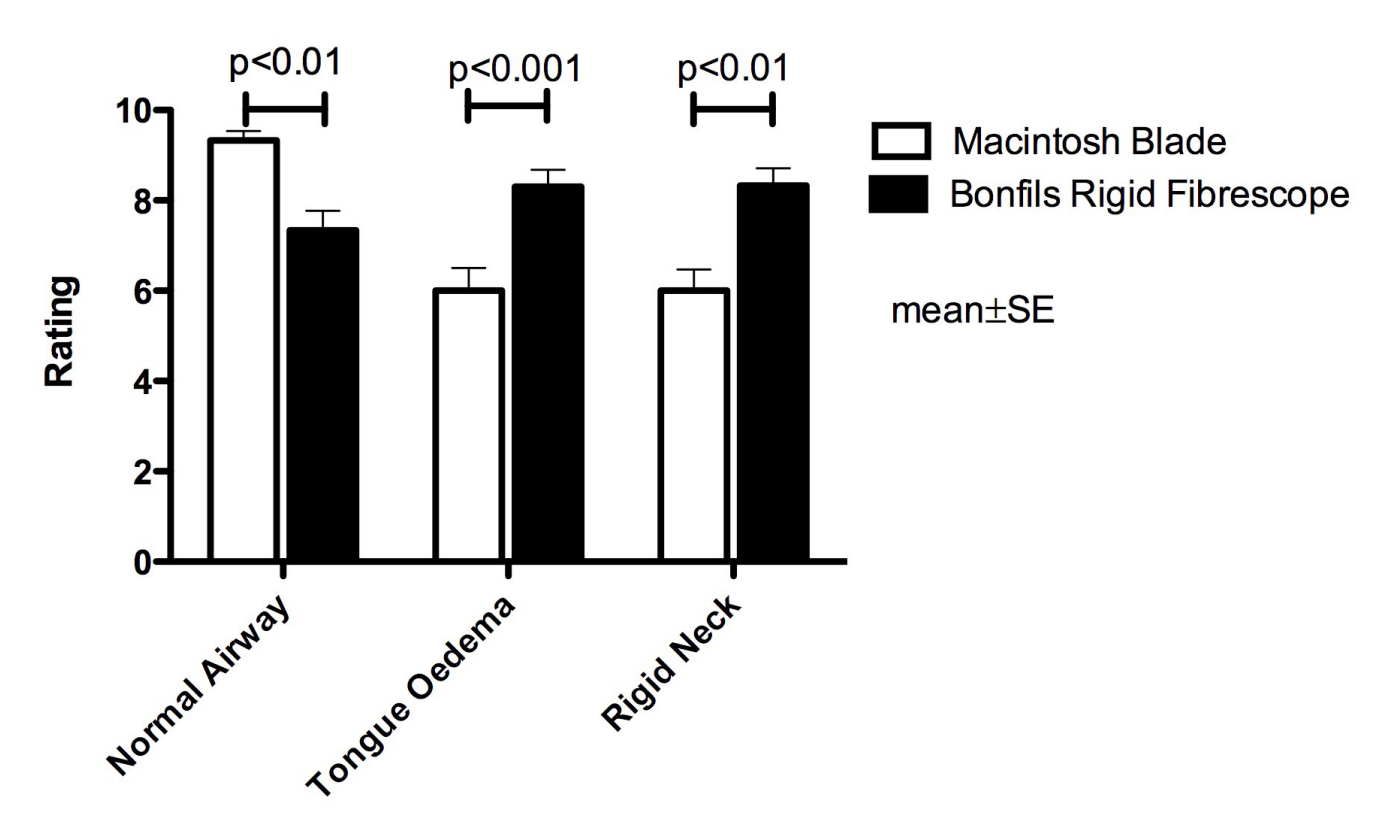
**Rating of both techniques in the three scenarios**. 10 = extremely easy, 0 = extremely difficult.

All physicians successfully intubated the trachea using the Macintosh laryngoscope in the normal airway situation. In contrast, 82% (25/30) of intubation attempts using the Bonfils succeeded (p < 0.05) (figure 2). The duration of the intubation attempts using the Bonfils was longer compared to the Macintosh laryngoscope (p < 0.001) (figure 3). The participants rated the use of the Macintosh laryngoscope as less difficult in this scenario (p < 0.01) (figure 4).

### Tongue oedema

In this scenario, 20/30 attempts (67%) to intubate the trachea using the Macintosh blade were successful. The success rate using the Bonfils was 83% (25/30) (p > 0.05; figure 2). The duration of tracheal intubation was similar with both devices. In the tongue oedema scenario, physicians ranked the Bonfils easier to use compared to the Macintosh laryngoscope (p < 0.001).

In comparison to the normal airway scenario, the time needed for intubating the trachea using the Macintosh laryngoscope was longer (p < 0.05) (figure 3) and rated more difficult (p = 0.001). The use of the Bonfils was rated less difficult (p < 0.05) in this difficult airway scenario (figure 4).

### Decreased cervical range of motion with jaw trismus

No difference was noted in the number of successful attempts (84% vs. 76%) (figure 2) and their duration (figure 3) using the Macintosh laryngoscope and the Bonfils, respectively. The time needed to intubate the trachea using the laryngoscope was longer compared to intubation with normal airway (p < 0.05, figure 3). In this scenario, the Bonfils was rated easier to use compared to the Macintosh laryngoscope (figure 4; p = 0.001).

Within the groups, the use of the Bonfils was rated easier (p < 0.05) and the use of the Macintosh laryngoscope more difficult (p = 0.001, figure 4) in comparison to the normal airway scenario.

## Discussion

The Bonfils rigid fibrescope is a device that is designed to assist in establishing an airway when direct laryngoscopy is difficult or fails. *Bein *described a high success rate after in-hospital failed direct laryngoscopy in trained users [13]. But until now, the learning curve of the Bonfils is not well evaluated. *Halligan *studied the learning curve of the Bonfils in two anaesthesiologists. His results suggest that a performer becomes proficient after 20 to 25 intubations [14]. According to *Byhahn*, 10 intubations supervised by an instructor seem to prove effective [10]. We show that physicians untrained in the use of the device have poor rate of successful intubations with first attempt in simulated normal airway situations. However, the success rate is similar between simulated normal and difficult airway situations. Our data suggest that the Bonfils might be an alternative device in the management of difficult intubation with at least the same rate of successful intubations than the Macintosh laryngoscope.

Various anatomical or pathologic conditions contribute to difficult airway management situations. Therefore, we simulated two potentially difficult airway scenarios. The simulation of different intubation scenarios has been widely used for similar studies in the past [15-17]. Although manikin studies have proven a reliable surrogate in the clinical context, study settings cannot entirely simulate the conditions of an emergency airway management scenario. The Bonfils requires a minimal retropharyngeal space to allow orientation according to anatomical landmarks of the fibrescope under direct vision. In humans it is necessary to create a sufficient retropharyngeal space to successfully visualize the vocal cords with the Bonfils fiberscope. This can be achieved with either a jaw thrust manoeuvre, with a laryngoscope blade or by lifting the base of the tongue with the index finger of the non-operating hand. Quick and sufficient endotracheal intubation is almost impossible if a retropharyngeal space is not created using one of these techniques.

A wide variation of results have been described between different manikins when airway devices have been tested [18]. While the SimMan's airway is generally considered very realistic, endotracheal intubation in any kind of manikin in general seems to be easier than in a real patient [19]. One reason is that tongue of the manikin is rigid and not as flaccid as in unconscious patients. Therefore a sufficient retropharyngeal space is present and it is not necessary to use a special technique while using the Bonfils in a manikin setting.

A typical situation which can lead to a difficult endotracheal intubation is the tongue oedema. In this scenario handling and use of the Bonfils was rated to be easier than the more common Macintosh laryngoscope. Although all physicians were skilled in the use of the Macintosh laryngoscope the failure rate was – from a patient's point of view – unacceptably high. Although the failure rate between both devices was statistically not significant, it is of clinical relevance. Despite their experience and familiarity with the Macintosh laryngoscope, the participants favoured the Bonfils. Due to the randomized order of the scenarios a potential learning curve using the Bonfils was not considered. The advantage of the Bonfils in the tongue oedema scenario is its small diameter enabling it to be manoeuvred even in minimal space. In theory a space of the outer diameter of the laryngeal tube seems to be adequate to perform successful intubation. Furthermore, the picture from its tip is being transfered to the physician's eye. Direct visualization of the glottis – a prerequisite using direct laryngoscopy with a Macintosh laryngoscope – is not required.

An attached camera with a monitor system allows an easier and less time consuming endotracheal intubation, especially in untrained physicians. However, endotracheal intubation was performed in this study by using the eyepiece of the Bonfils. A major limitation of the video unit is its portability. Therefore, the use of a video system in prehospital emergency medicine and emergency intubation can not be suggested for routine use.

The cervical spine has to be immobilized by a rigid collar until a potential injury has been ruled out in the hospital. The limited mouth opening and the missing neck extension is associated with a Cormack and Lehane grade 3 and 4 view [20]. We used a decreased cervical range of motion with jaw trismus in the manikin to simulate the airway situation of trauma patients with a rigid collar. No difference was evident between both devices in respect to the failure rate and duration of the attempts. However, the participants of this study favoured the Bonfils. It is likely that the main reason for this finding is that endotracheal intubation using the Bonfils can be achieved using less strength and without any cervical spine movement. This potential benefit of the Bonfils is in accordance with previously published manuscripts [21,22]. Several cases of successful pre-hospital endotracheal intubation using the Bonfils in patients with an immobilized cervical spine exist [10]. The small diameter of the Bonfils is in this scenario the advantage, too. It requires only minimal mouth opening to advance the tip into the pharynx [23].

The use of a flexible intubation fibrescope is a common device to secure the difficult airway [24]. However, the insertion of an intubation fibrescope is more difficult and time consuming in patients with immobilized cervical spine than in the normal patient and in emergency situations the endotracheal intubation attempt without interposed mask ventilation should not exceed 30–40 s [1].

Though the failure rate of the Bonfils was less compared to the Macintosh laryngoscope in the decreased cervical range of motion with jaw trismus scenario, we do not recommend using the Bonfils in cases of difficult airway management without extensive training and clinical routine. As the success of emergency airway management is strongly influenced by the level of training and expertise with the device used, it should be suitable for use in daily practice.

## Conclusion

Our data suggests that in comparison to the Macintosh laryngoscope the use of the Bonfils in a normal airway is more difficult and time consuming at least if the physicians are not experienced with the Bonfils device. These data indicate that physicians must undergo an intensive training of normal and difficult airway management scenarios to improve rate of successfully securing the airway in these critical situations.

## Competing interests

Karl Storz GmbH & Co. KG, Tuttlingen, Germany, provided the fiberscope used in this study. The Departments of Anaesthesiology in Mainz and Idar-Oberstein are supported by Karl Storz GmbH & Co. KG, Tuttlingen, Germany.

## Authors' contributions

TP and RRN contributed equally to this article. TP has made substantial contributions to conception, acquisition of data and drafting the article. RN has made substantial contributions to analysis, interpretation of data and in drafting the article. FH has made substantial contributions to analysis and interpretation of data. CW has made substantial contributions to conception and revised the manuscript critically for important intellectual content. AT has made substantial contributions to conception, acquisition of data and revised the manuscript. All authors read and approved the manuscript.
